# On Gastro-Jejunostomy for Pyloric Stenosis, Gastric Ulcer, and Some Other Non - Malignant Conditions

**Published:** 1904-05-21

**Authors:** 


### ON GASTRO-JEJUNOSTOMY FOR PYLORIC STENOSIS, GASTRIC ULCER, AND SOME OTHER NON-MALIGNANT CONDITIONS.

Mr. Gilbert Barling, discussing this operation, classifies the conditions for which he has performed the operation into three groups:—(1) In pyloric or duodenal stenosis, and hour-glass stomach, for providing relief to gastric stasis. (2) To put the stomach at rest and thus relieve hæmorrhage and pain, while at the same time promoting the healing of ulcers. (3) For atonic dyspepsia, to provide a passive opening from the stomach, situated at the most dependent part of the dilated organ. In chronic dyspepsia, when medical treatment has failed, this operation has been performed as a last resource. In simple uncomplicated stenosis of the pylorus or hour-glass contraction the relief has been almost always complete; the more severe the symptoms, the more marked the benefit.

Hæmorrhage and pain have both been relieved; in two cases this was not permanent, both died, one of severe hæmorrhage, the other of perforation; in each instance the ulcer was in the duodenum. In one case of atonic dyspepsia the stomach remained dilated after operation, but the vomiting ceased. A case of chronic indigestion was not relieved by operation. The earlier recognition of gastric stasis leading to earlier relief by surgical measures should be aimed at. Early operation in this, as in so many other conditions, means safe operation.

With regard to the method employed, stretching the stricture (pylorodiosis) should be abandoned as efficient dilation risks splitting the peritoneal coat and causing peritonitis. Pyloroplasty is a much better operation, but not preferable to gastro-jejunostomy. The stomach may be united by its anterior or posterior surface to the jejunum, and by simple suture or with the aid of a bobbin or button. The anterior operation may be followed by regurgitation of bile into the stomach, owing to the displacement of the jejunum and to the drag on the transverse colon. Posterior anastomosis by bobbin or button seems most satisfactory for the reasons that the operation is quickly and easily performed, and there is little regurgitation; but the bobbin is an inconvenient object to suture round, and the button leaves a possible source of obstruction and ulceration in the intestine. Mr. Barling has performed the operation 18 times; by suture eight times, all recovered; by bobbin twice, with one death; by Murphy's button eight times, with one death. The first death was from hæmorrhage without discoverable cause. The second was due to the button ulcerating through the junction on the ninth day after operation. In four out of the eight cases of suture troublesome vomiting occurred, and in one a secondary anastomosis between the portion of the jejunum above and that below the union with the stomach was necessary to save the patient's life. In the other cases troublesome vomiting did not occur. As to the subsequent potency and efficiency of the opening, it appears to matter little how the union is effected.

When preparing the patient for operation, nothing but milk is given for 48 hours previously. Nutrient enemata are given every four hours after six o'clock on the evening preceding the operation.

Stomach washing, because disagreeable, is never resorted to, and the stomach and intestine have in 9 cases out of 15 been free from bacterial life. The abdomen is freely opened in the middle line, the omentum and transverse colon turned up, the transverse mesocolon divided, thus exposing the stomach. A portion of jejunum, not less than 12 inches from its junction with the duodenum is selected, emptied, and clamped with Doyen's forceps. A pouch of stomach drawn through the opening in the mesocolon is similarly treated, and the parts packed round with sponges. The junction is made by two rows of suture; the outer, including peritoneum and muscle, is interrupted once when it has completed half a circle and then laid aside. The stomach and jejunum having been opened and the vessels ligatured, the second suture is applied, including all the coats, and makes a complete uninterrupted circle. The clamps are removed by the assistant who has been holding the parts in apposition, and the outer row of suturing is resumed and completed. The parts are swabbed with saline lotion and returned. Nothing is given by the mouth for a few hours, then sips of hot water, and after 24 hours milk and water—a tablespoonful every half hour. On the fourth day, beef tea and custard are added to the diet. On the tenth day minced meat is allowed, and ordinary diet may be resumed at the end of three weeks.

